# Tiny Intensifiers: Nanoparticles Worsen Lung Effects of Bacterial Endotoxin

**Published:** 2006-09

**Authors:** Victoria McGovern

Exposure to particulate matter in the air, especially extremely fine particles, has been associated with increased morbidity and mortality from lung and cardiovascular disease. Effects grow more significant with decreasing particle size. However, the size-related effects of nanoparticles—particles less than 100 nm (0.1 μm) in diameter—on pulmonary inflammatory conditions have not been fully investigated. This month a team of Japanese investigators reports that nanoparticles can increase lung inflammation associated with bacterial endotoxin, or lipopolysaccharide (LPS) **[*EHP* 114:1325–1330; Inoue et al.]**.

Inhalation of particles with a mass median aerodynamic diameter of 10 μm or less is associated with increased hospitalization for asthma, bronchiolar irritation, and lower respiratory tract infections, while exposure to particles 2.5 μm and smaller, including common carbon-cored pollutants from diesel exhaust, exhibit a stronger epidemiological link to death from cardiopulmonary and respiratory effects. Particles even smaller, 0.1 μm or less, are thought to move beyond the respiratory system, perhaps reaching the blood stream. The tiniest particles are not just smaller than other pollutants; they have more surface area for a given weight—imagine the difference between the surface area of a solid glass cube compared to that of an equal weight of fine glass beads. Both the small size of nanoparticles and the high surface area that they present to cells may contribute to their effects.

For the current study the researchers used ultrafine carbon black, a form of elemental carbon used in the printing industry, to explore how exposure to nanoparticles impacted antigen-related inflammation of airways in mice. Using carbon black formulations with diameters of 14 nm and 56 nm, they looked at how LPS’s effects changed in the context of nanoparticles in the airway.

The effects of the nanoparticles by themselves was slight, while exposure to LPS alone increased by 12-fold the number of cells harvested by bronchoalveolar lavage (a measure of airway inflammation). However, simultaneous exposure to LPS and to 14-nm particles amplified the effect to yield a 20-fold increase. Simultaneous exposure to LPS and to 56-nm particles resulted in a smaller, statistically insignificant boost in the effects of LPS.

It was not just cell infiltration that was affected. Histology showed that lung exposure to a mixture of 14-nm particles, which had only minor effects themselves, and LPS led to recruitment of neutrophils in the parenchyma, the actual respiratory surface of the lung. LPS-driven oxidative stress and expression of chemokines and cytokines also were amplified in the presence of these small particles, and changes in blood coagulatory factors were seen as well. The larger 56-nm particles increased the effects of LPS in some but not all assays.

Taken together, these observations suggest that ultrafine carbon-cored particles, perhaps including those present in vehicle exhaust, can make respiratory damage from commonly encountered bacterial endotoxins even worse.

## Figures and Tables

**Figure:**
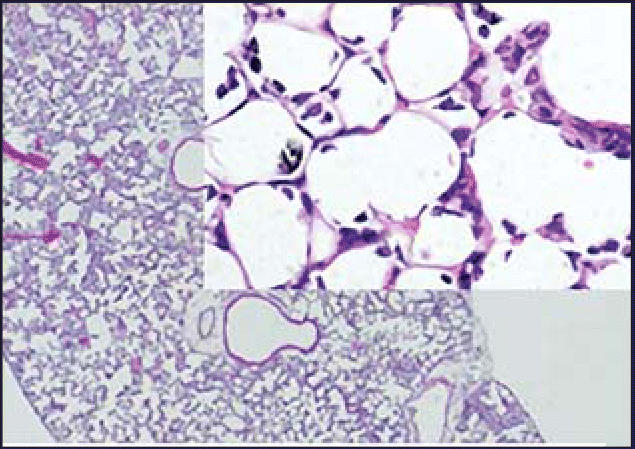
Nanoparticles in inflamed lung tissue

